# Identification of the Aggregation Pheromone of the Melon Thrips, *Thrips palmi*

**DOI:** 10.1371/journal.pone.0103315

**Published:** 2014-08-07

**Authors:** Sudhakar V. S. Akella, William D. J. Kirk, Yao-bin Lu, Tamotsu Murai, Keith F. A. Walters, James G. C. Hamilton

**Affiliations:** 1 Centre for Applied Entomology and Parasitology, School of Life Sciences, Huxley Building, Keele University, Keele, Staffordshire, England, United Kingdom; 2 Institute of Plant Protection and Microbiology, Zhejiang Academy of Agricultural Sciences, Hangzhou, Zhejiang, China; 3 Laboratory of Applied Entomology, Faculty of Agriculture, Utsunomiya University, Utsunomiya, Tochigi, Japan; 4 Food and Environment Research Agency, Sand Hutton, York, North Yorkshire, England, United Kingdom; INRA-UPMC, France

## Abstract

The objective of this study was to identify the aggregation pheromone of the melon thrips *Thrips palmi*, a major pest of vegetable and ornamental plants around the world. The species causes damage both through feeding activities and as a vector of tospoviruses, and is a threat to world trade and European horticulture. Improved methods of detecting and controlling this species are needed and the identification of an aggregation pheromone will contribute to this requirement. Bioassays with a Y-tube olfactometer showed that virgin female *T. palmi* were attracted to the odour of live males, but not to that of live females, and that mixed-age adults of both sexes were attracted to the odour of live males, indicating the presence of a male-produced aggregation pheromone. Examination of the headspace volatiles of adult male *T. palmi* revealed only one compound that was not found in adult females. It was identified by comparison of its mass spectrum and chromatographic details with those of similar compounds. This compound had a structure like that of the previously identified male-produced aggregation pheromone of the western flower thrips *Frankliniella occidentalis.* The compound was synthesised and tested in eggplant crops infested with *T. palmi* in Japan. Significantly greater numbers of both males and females were attracted to traps baited with the putative aggregation pheromone compared to unbaited traps. The aggregation pheromone of *T. palmi* is thus identified as (*R*)-lavandulyl 3-methyl-3-butenoate by spectroscopic, chromatographic and behavioural analysis.

## Introduction

Thrips are small insects, typically only 1–2 mm long, belonging to the order Thysanoptera. Adults and larvae of many species cause serious commercial damage to crops grown in protected environments, such as glasshouses and polytunnels (tunnels covered with polythene), and also to open-field crops, through feeding and virus transmission. Most commercially important thrips pest species are in the genera *Thrips* and *Frankliniella*, which belong to the same sub-family (Thripidae: Thripinae).

The western flower thrips *Frankliniella occidentalis* (Pergande) [1], [2] and *Frankliniella intonsa* (Trybom) [3] have male-produced aggregation pheromones that are attractive to both female and male conspecifics. *F. occidentalis* males form lek-like aggregations within which there are aggressive male–male interactions. Females arrive continually, mate, and leave immediately, so although both sexes arrive at the aggregations, they contain predominantly males [4]. The aggregation pheromone is probably used by males and females to locate these mating aggregations [2]. In *F. occidentalis*, the pheromone has been tested in the field and identified as a single component, the monoterpene ester neryl (*S*)-2-methylbutanoate (N(*S*)2 MB) [2]. In *F. intonsa* the aggregation pheromone may be a two-component mix of N(*S*)2 MB with (*R*)-lavandulyl acetate ((*R*)LA) [3], but the effects of synthetic compounds have not yet been tested.

These pheromone components probably originate from glandular tissue underlying a series of structures on the underside of the abdomen of adult males, known as sternal pore plates [5]–[7]. Species in the genus *Thrips* also have male pore plates [6] and aggregations of males have been recorded in some species [8], leading to speculation that they may also produce an aggregation pheromone.

The melon thrips *Thrips palmi* Karny is a global pest of a wide range of plants, particularly in the Solanaceae and Cucurbitaceae, including important vegetable and ornamental crops such as eggplant (brinjal, aubergine), melon, cucumber, sweet pepper and chrysanthemum [9], causing significant damage both by feeding and as a vector of tospoviruses [10]. Since the late 1970s, it has spread around the world, probably originating from southeast Asia, and is now a pest across Asia and the Pacific and is also found in Florida, the Caribbean and parts of South America, Africa and Australia [9], [11]. It has recently been recorded for the first time in Iran [12]. Although *T. palmi* is not currently a problem in Europe, its well documented global dispersal in association with the international trade in plants or plant products has added an extra dimension to its pest status, and it is considered to pose a considerable threat to the European horticulture industry [13], [14]. It is commonly intercepted at points of entry on imports of cut flowers, fruit and vegetables throughout the world, and such dispersal pathways result in crop colonisation. Its behaviour results in it seeking out small enclosed spaces, which can make it difficult to detect. Short generation times result in rapid population increases and development of insecticide resistance can result in control failures. Some incursions into European crops have occurred, where outbreaks are subject to plant quarantine legislation, and have been successfully eradicated [9], [15], but this has been more readily achieved if management actions commence soon after initial infestation when populations are small. Enhanced methods for early detection and control are thus of central importance to maintain biosecurity and enhance the effect of current control methods.

The objectives of this study were to identify any male-produced volatile compounds of *Thrips palmi* and test whether they act as an aggregation pheromone in the field, with a view to providing a tool for potential use in both commercial and quarantine pest detection and management.

## Materials and Methods

### Thrips

Field experiments and some thrips collections were carried out on private land and we confirm that the owner of the land gave permission to conduct the study on this site.

The thrips for olfactometer bioassays were reared from *T. palmi* collected from an eggplant crop (*Solanum melongena* L.) in Hangzhou, Zhejiang Province, China (N 30° 18.331′ E 120° 11.730′). They were reared on bean pods (*Phaseolus vulgaris* L.) in 4.5 L glass canning jars at 27±1°C, 65–75% r.h., 16∶8 light:dark. Mixed-age adult thrips were collected arbitrarily from the colony. To obtain known-age virgin females, large numbers of second-instar larvae were collected from the colony and transferred individually into 0.5 ml microcentrifuge tubes containing a section of bean pod. They were examined daily and virgin females were used for the experiments 1–3 d after emergence.

All adult male and female *T. palmi* used for the collection of volatile chemicals were obtained from the leaves of commercial eggplant crops (*S. melongena* var. Senryo 2) grown in a polytunnel at Himuro near Utsunomiya, Tochigi Prefecture, Japan (N 36° 30.483′ E 139° 59.536′).

To identify any volatile compounds that might be produced exclusively by male *T. palmi*, the headspace volatiles of both adult males and females with appropriate controls were collected separately and analysed. *T. palmi* were transported from Utsunomiya University, Japan, in small plastic boxes (12 cm×8 cm×4 cm) lined with layers of moistened tissue on fresh sprouting broad bean seeds (*Vicia faba* L.) to the Central Science Laboratory (CSL) (now Fera), York, UK. The insects were held in secure quarantine facilities (license number: PHL 251B/5328(02/2006) amended (04/2006)) at 23°C, 65% r.h., 16∶8 light:dark until required.

Additional collections of adult male and female *T. palmi* were made in the field in Japan in August 2011 and again in October 2011 at Himuro for entrainment of headspace volatiles. These additional entrainments were undertaken on mixed male and female groups of *T. palmi* with the aim of allowing us to collect and store larger quantities of the target compound that was already identified from our solid phase micro extraction (SPME) entrainments at CSL. The additional material allowed us to carry out further comparisons with mass spectrometry (MS) data held in the coupled gas chromatography/mass spectrometry (GC/MS) library as well as further chiral and achiral chromatography. Collections were made between 10∶00–16∶00 h. Adults were aspirated from the eggplant leaves and held in clean glass containers (50 ml round bottomed (r.b.) flasks) and kept cool in an ice box. Approximately 1 g of eggplant leaves and petals were added to the r.b. flasks to provide a food source and maintain humidity levels until the thrips were used for experimentation. Four separate collections were made with the first containing approximately 35 males and 400 females, the second 20 males and 365 females, the third 26 males and 415 females and the fourth 18 males and 300 females. The imbalance of the sexes was because males are found much less frequently than females in the field.

To confirm that the collected thrips were *T. palmi*, representative samples were checked under a stereo microscope in the UK and Japan. The main characteristic features are: body colour yellow to white, antennae 7-segmented, macropterous with wing-vein setae interrupted, ocelli red, and ocellar setae III outside the ocellar triangle [16]. Females are distinguished from males by the pointed shape of the tip of the abdomen and the presence of an ovipositor. Males have no ovipositor and the tip of the abdomen is blunt.

### Olfactometer Bioassays

The response of adult thrips to male- or female-produced volatiles was tested in a glass Y-tube olfactometer. This had a stem 60 mm long, two arms 60 mm long, separated from each other at an angle of 90°, and an internal diameter of 5 mm. Air, filtered through activated charcoal, humidified and split into two air streams, each of which was fed through a 50 ml glass flask and into one arm of the olfactometer was drawn through at a flow rate of 60 mm/s. The two flasks provided test and control odour (clean air) sources. The flasks were illuminated from above by four flourescent tubes and by one arm of a fibre-optic cold-light source at a distance of 40 mm from the Y-tube (total illumination was approximately 10,000 lux). Connections between the components of the olfactometer apparatus were made with Teflon tubes. Olfactometer experiments were carried out at 25±2°C. Forty mixed-age adult thrips were collected with a small aspirator, anaesthetized with carbon dioxide, the sex of individuals was checked under a microscope, and they were then transferred into the treatment flask as the odour source. Test thrips were transferred individually to the stem of the Y-tube with a fine brush. Each thrips was observed for a maximum of 3 min, and its choice for one of the two odour sources (treatment or control) was recorded when it crossed a line 20 mm down either arm. ‘No choice’ was recorded if the line was not crossed after 3 min. After five thrips were tested, odour sources entering the arms of the Y-tube were swapped to avoid any potential bias in the apparatus. Each odour comparison was repeated four or five times on different days, with a total of 15–20 thrips per day. The apparatus was cleaned before each test by rinsing with hexane and baking in an oven (200°C).

The data were analysed with IBM SPSS Statistics 19 (IBM Corp., USA). Responses were tested by a binomial test with exact two-tailed *P* values, with the null hypothesis that the two arms were chosen with equal probability. “No choices” were excluded from the analysis.

### Headspace Volatile Collection

All glassware used in the collection of headspace volatiles was cleaned by first washing in a 5–10% detergent solution, then rinsing with distilled water, drying with acetone and finally heating at 200°C in a clean oven overnight to remove potential contaminants. Teflon tubing used in the portable entrainment apparatus was cleaned by first washing in a 5–10% detergent solution, rinsing with distilled water, drying with acetone and then leaving in a fume hood at room temperature overnight to allow solvent to evaporate fully.

### Entrainments at CSL

*T. palmi* (males, females or larvae) were removed from the sprouting beans with a small aspirator, anaesthetised with carbon dioxide, and transferred into a clean glass container (volume 1.9 ml) that was then sealed with Teflon tape. The thrips were illuminated from above with a 60 W tungsten filament lamp to induce patrolling behaviour [1]. Headspace volatiles were collected on a divinylbenzene (DVB)/carboxen/polydimethylsiloxane (PDMS) SPME fibre assembly (57348-U, Supelco, Poole, UK) inserted into the glass container containing the thrips through the Teflon tape at 27°C for 4–18 h [1]. The numbers of males, females and larvae entrained in this way varied from 30 to 100 per replicate and in total four entrainments of each sex and stage were carried out. The following entrainments were carried out, males by themselves, females by themselves and larvae. As the males, females or larvae were removed from the bean sprouts and entrained away from this food source a separate SPME entrainment of bean sprouts was not done. After each entrainment the SPME fibre was sealed in a clean glass tube and transferred to Keele University for GC/MS analysis.

### Entrainments in Utsunomiya

Entrainments of headspace volatiles were carried out in Utsunomiya to provide greater quantities of the male-specific compound identified by SPME entrainment of *T. palmi* at CSL described above. After field collection the 50 ml r.b. flasks containing the thrips were transferred to the laboratory and the headspace volatiles collected using a portable entrainment apparatus (Barry Pye, Kings Walden, Herts. UK). Air, pushed through the entrainment apparatus by a pump, was first cleaned by passing it through an activated charcoal filter and then into a r.b. flask containing the thrips and plant material (for the thrips to feed on) via a Drechsel head. A control entrainment of eggplant only was also carried out. The air exiting from the r.b. flask then passed into a glass column containing an adsorbent polymer (ORBO 402, Tenax-TA). All tubing and components within the entrainment apparatus were connected with Swagelok connectors or Teflon tubing joints and were sealed with Teflon tape (Sigma-Aldrich Company Ltd., Gillingham, UK) to eliminate leakage of air. Air flow at the outlet of the Tenax-TA tube was measured with a bubble flow meter and maintained at 5 ml/s by adjustment of a rotameter (GPE Ltd., Leighton Buzzard, UK) at the air inlet side of the apparatus.

The entrainment was run continuously for a period of 4 days. The Tenax-TA columns were replaced every 24 h with a fresh adsorbent column when fresh petals were also added to both r.b. flasks.

Volatiles were eluted from the Tenax-TA tubes using 2 ml of a 95∶05 mixture of *n*-hexane (SupraSolv grade; Merck, Germany) and ethyl acetate (Chromatography/HPLC grade; Fisher Scientific, Loughborough, UK). The extracts from the four collections were concentrated under a gentle stream of air to 1 ml and returned to Keele University where they were combined and the volume reduced again to 100 µl for GC/MS analysis. The amount of monoterpene ester present in the *T. palmi* extracts from Japan was quantified by comparison of the peak area of the unknown ester with a known amount of neryl (*S*)-2-methylbutanoate by GC/MS analysis.

### Coupled Gas Chromatography/Mass Spectrometry

GC/MS analyses were carried out on either a HP 5890 II+ GC coupled to a HP 5972A MS or an Agilent 7890 GC coupled to an Agilent 5973 MS (Agilent Technologies, Ipswich, UK). The 5972A was operated in electron impact (EI) (70 eV, 180°C) mode only. The 5973 instrument was operated in either EI (70 eV, 180°C) or chemical ionization (CI) mode. CI analyses were carried out using isobutane as the reagent gas.

For the 5890 GC the carrier gas was helium (1 ml/min) and the injector was a Merlin Microseal (Thames-Restek, High-Wycombe, UK) septum-less heated injector (180°C) fitted with a SPME glass injection sleeve (0.75 mm i.d.; Supelco). SPME samples were injected in the splitless mode and desorbed for 8 min before the fibre assembly was withdrawn. Non-SPME samples (≤1 µl) of *T. palmi* extracts were also injected via this injector set in the splitless mode using a standard 10 µl syringe (Sigma Aldrich, UK) to maintain sensitivity. An initial temperature of 40°C was held for 2 min, increased (10°C/min) to 120°C, then increased (6°C/min) to 180°C and then increased (10°C/min) to the final temperature of 250°C (held for 1 min). The MS transfer line was set at 280°C. Prior to each SPME thrips entrainment analysis, a blank fibre was analysed to check system performance for the presence of possible contamination.

For the 7890 GC the injector was a multimode inlet set in splitless mode at 180°C and the GC analytical conditions were as described above for the 5890 GC.

SPME-collected headspace volatiles, hexane:ethyl acetate extracts of Tenax-TA entrainment tubes and synthetic standards were analysed on both HP5MS (Supelco), DBWax (Supelco) and chiral CycloSil-B (Agilent J&W, Agilent, Wokingham, UK) fused silica analytical columns (30 m×0.25 mm i.d., 0.25 µm phase thickness) as appropriate.

The retention index (RI) of the *T. palmi* compound was calculated relative to the retention times of saturated hydrocarbons. The RI and mass spectrum of the *T. palmi* compound were then compared against the RIs and mass spectra of a library of 200 synthetic monoterpene C5 esters that included pentanoates (72 compounds), pentenoates (83 compounds), pentadienoates (12 compounds) and pentynoates (33 compounds). The library was prepared by synthesising 200 of the possible combinations of 19 C5 fatty acids and their isomers with 17 commercially available acyclic, monocyclic and bicyclic monoterpene alcohols and their isomers. The esters were then analysed individually according to the general methodology described below and RI and EI/MS data were collected for each compound and isomer on both DB5 and DBWax columns. RI and EI/MS data were also collected for molecules with chiral centres on a CycloSil-B column.

### Chiral Chromatography of Monoterpene Esters

Analysis of the enantiomeric composition of synthetic standards and authentic thrips material was carried out on the Agilent 7890 coupled 5972 GC/MS with a CycloSil-B column. The carrier gas was helium (flow rate 1 ml/min). Samples were introduced via a heated multimode injector port (180°C) and the GC was temperature programmed with an initial 2 min at 55°C, an increase of 5°C/min to 115°C, held for 1 min, then an increase of 0.5°C/min to 165°C.

### Chiral Chromatography of the Racemate and R and S enantiomers of Lavandulol (5-Methyl-2-(1-methylethenyl)hex-4-en-1-ol)

The GC was temperature programmed with an initial 2 min at 55°C, then an increase of 10°C/min to a temperature of 125°C, held for 1 min, then an increase of 5°C/min to a temperature of 200°C and then to the final temperature of 250°C (10°C/min). (*S*)-lavandulol eluted first at 19.86 min and (*R*)-lavandulol at 20.29 min.

### Synthesis of Racemic Lavandulyl 3-Methyl-3-butenoate

**Figure 1:**
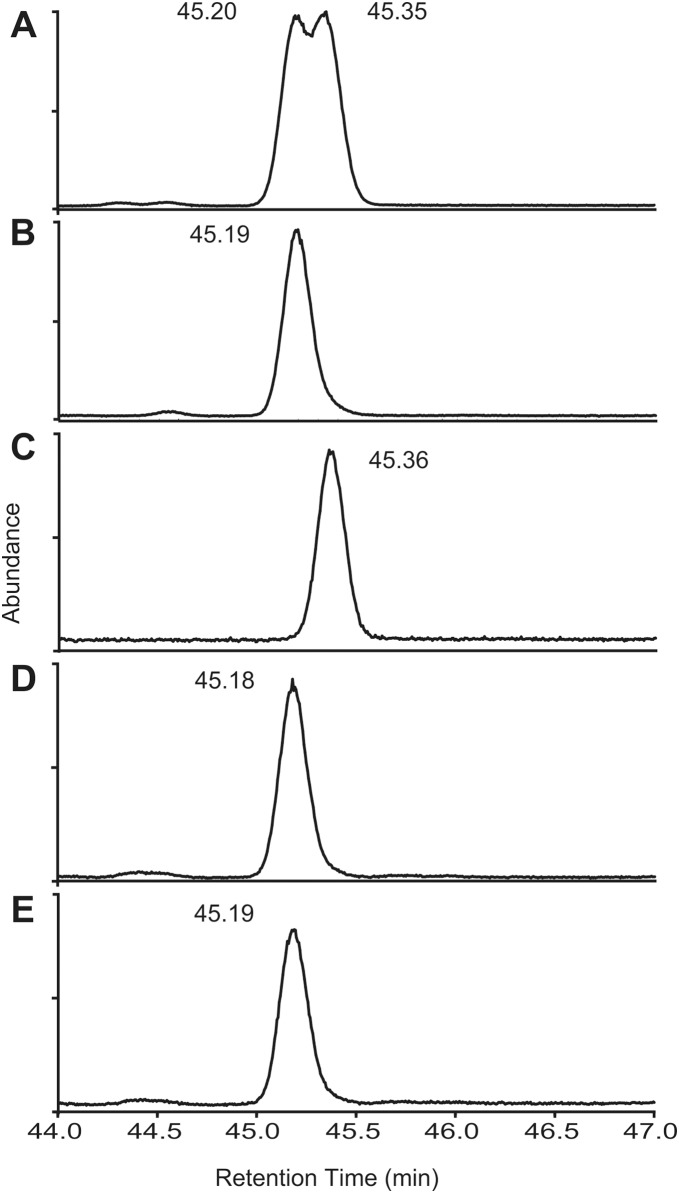
GC/MS analysis on a chiral column. Confirmation of *Thrips palmi* aggregation pheromone as the (*R*) enantiomer by GC/MS analysis on a CycloSil-B analytical column: (A) section of the TIC chromatogram from 44 to 47 min showing the two partly resolved peaks obtained from racemic lavandulyl 3-methyl-3-butenoate; (B) the peak obtained on injection of the (*R*)-lavandulyl 3-methyl-3-butenoate enantiomer; (C) the peak obtained on injection of the (*S*)-lavandulyl 3-methyl-3-butenoate enantiomer; (D) the peak obtained on injection of the *T. palmi* natural compound; (E) the enhanced peak obtained on co-injection of the *T. palmi* natural compound and (*R*)-lavandulyl 3-methyl-3-butenoate.

**Figure 2:**
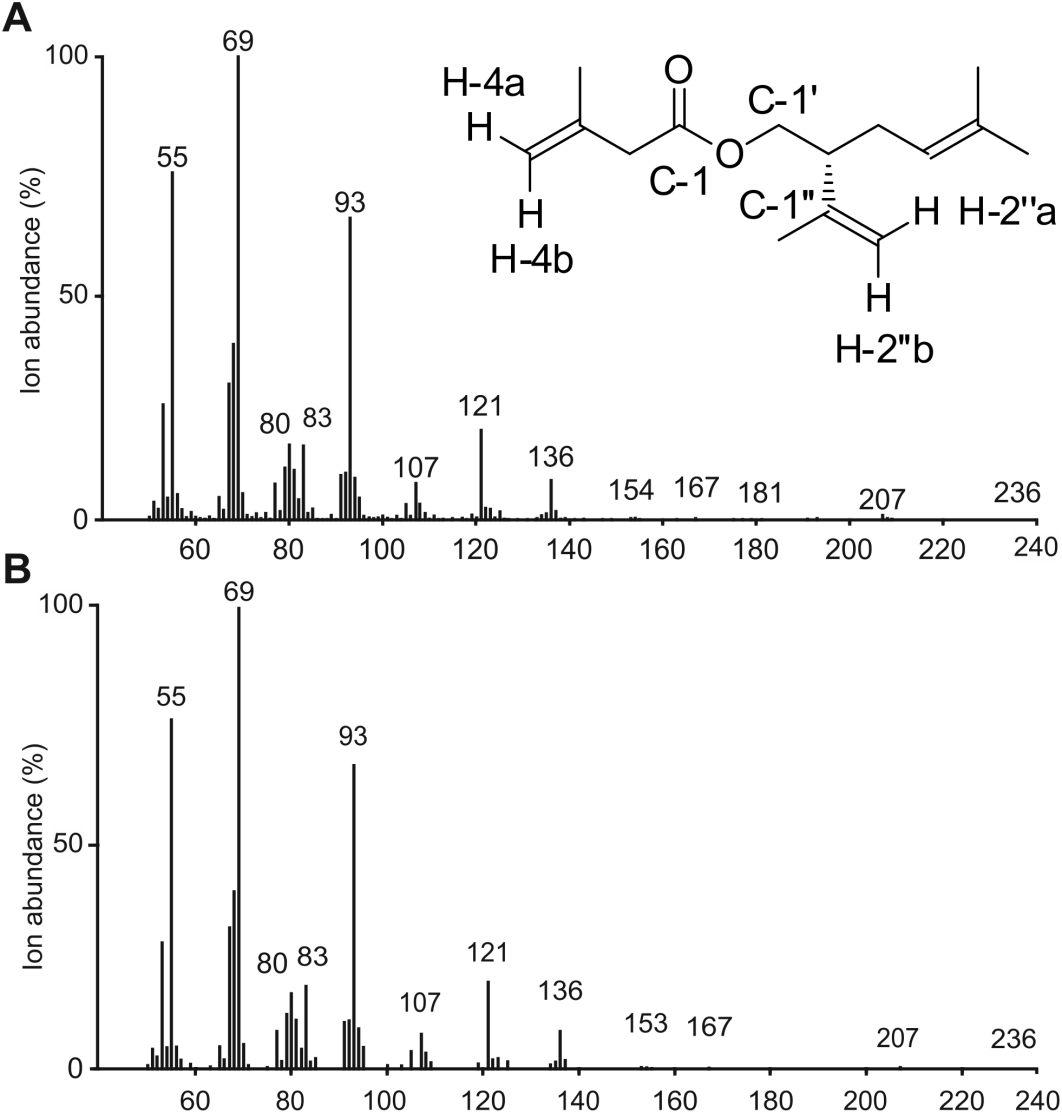
EI mass spectra of the major terpenoid component and the synthetic (*R*)-lavandulyl 3-methyl-3-butenoate. EI mass spectra (70 eV) of (A) the major terpenoid component (peak *a*) of the headspace volatiles of male *Thrips palmi* and (B) the mass spectrum of synthetic (*R*)-lavandulyl 3-methyl-3-butenoate. The inset shows the labelled structure of (*R*)-lavandulyl 3-methyl-3-butenoate.

Lavandulol (1 mmol; Sigma-Aldrich), 3-methyl-3-butenoic acid (1.2 mmol; Sigma-Aldrich), and 4-dimethylaminopyridine (DMAP) (0.05 mmol; Sigma-Aldrich) were dissolved in dry dichloromethane (2 ml), and the solution was stirred in an ice bath. N,N’-Dicyclohexylcarbodiimide (DCC) (1.2 mmol; Sigma-Aldrich) was added portionwise over 30 min, and stirring was continued for another 30 min with cooling and then for 3 h at room temperature. The N,N’-dicyclohexylurea reaction by-product was filtered off, and the precipitate was washed with petroleum ether. The filtrate was washed with saturated aqueous sodium bicarbonate solution, dilute hydrochloric acid, and water, dried over magnesium sulphate, and filtered. After concentration, the residue was purified by column chromatography on silica gel (40 g, 100–200 mesh) eluted with a mixture (98∶2) of petroleum ether:ethyl acetate. Pure fractions, identified by thin-layer chromatography, were collected and concentrated giving the ester in 95.3% yield and 100% purity. The (*R*) and (*S*) enantiomers of lavandulyl 3-methyl-3-butenoate were partially separated by analysis of the reaction product on a CycloSil-B column (Fig. 1A). ^1^H NMR (CDCl_3_, 300 MHz): δ 5.05 (t, 1H, J = 6.9 Hz, H-4a (hydrogen number by position on the structure, see Fig. 2) CH_3_C = CH), 4.90 (m, 1H,  = CH-CH_2_), 4.83 (m, 1H, H-4b CH_2_C = CH), 4.83 (m, 1H, H-2″b CH_3_C = CH), 4.70 (d, 1H, J = 0.8 Hz, H-2″a CHC = CH), 4.07 (dd, 2H, J = 7.5, 3.0 Hz, CH_2_O_–_COR), 3.02 (s, 2H, CH_2_C( = CH_2_)(CH_3_), 2.36–2.45 (m, 1H, CH-CH_2_O), 2.00–2.20 (m, 2H, CH_2_-CH-CH_2_O), 1.80 (br s, 3H, CH_3_^_^C = ), 1.69 (br s, 3H, CH_3_^_^C = ), 1.68 (br s, 3H, CH_3_^_^C = ), 1.60 (br s, 3H, CH_3_^_^C = ).^13^C NMR (CDCl_3_, 75 MHz) δ 171.45, 144.80, 138.59, 133.00, 121.57, 114.72, 112.52, 66.06, 46.10, 43.52, 26.55, 25.80, 22.50, 19.94, 17.84.

### Synthesis of (R)- and (S)-Lavandulyl 3-Methyl-3-butenoate

The (*R*) enantiomer of lavandulol was obtained from racemic lavandulol (97% purity, Fluka) by a lipase-catalysed acylation using porcine pancreas lipase type ll [17]. Enantiomerically enriched (*R*)-lavandulol was obtained (0.68 g) and chiral chromatography on CycloSil-B capillary column showed an enantiomeric excess of 98%.

The (*S*) enantiomer was prepared by alkaline hydrolysis of (*S*)-lavandulyl acetate [18]. Enantiomerically enriched (*S*)-lavandulol was obtained (5 mg) and chiral chromatography on the CycloSil-B analytical capillary column showed an enantiomeric excess of 98%.

The (*R*) and (*S*) lavandulyl 3-methyl-3-butenoate esters were prepared separately by esterification of the alcohol with 3-methyl-3-butenoic acid as described for the racemic lavandulol above. Both esters were then purified by column chromatography. The formation of both the (*R*) and (*S*) enantiomers was shown by analysis of the products by chiral chromatography on the CycloSil-B column (Fig. 1B and 1C).

### Field Trials

The biological activity of the male-produced compound was tested in September 2012 in the same greenhouse (a polytunnel 50 m long×5 m wide×2.5 m high at the apex) near Utsunomiya, Japan that had been used in August and October 2011 to collect male and female *T. palmi* for headspace volatile analysis. The effect of the chemical was tested by comparing the number of thrips caught on traps with and without the synthetic aggregation pheromone in a crop of mature eggplant (*Solanum melongena* variety ‘Senryo 2’) with two rows of crop running along the length of the greenhouse.

Rectangular blue sticky traps, 10 cm×25 cm (Takitraps, Syngenta Bioline, UK), were suspended on wire hangers so that they were directly above the middle of the row and placed so that the base of the trap was about 10 cm above the canopy of the crop, which was at a height of about 1.2 m. Blue traps were used because they are widely reported as being highly attractive to *T. palmi* and thus the additional effect of the putative pheromone over that of an already very attractive trap would be tested [19]. Pre-sampling of the crop indicated that it was infested with two species of thrips: *T. palmi* and, to a lesser extent, *F. intonsa*. The paper protecting the north-facing side of each trap was removed to expose the sticky surface and a rubber septum (diam. 6.3 mm, length 10.8 mm, pre-cleaned, International Pheromone Systems Ltd., Deeside, UK) was stuck to the middle of the exposed side. The test septa were loaded with 30 µg of (*R*)-lavandulyl 3-methyl-3-butenoate in 30 µl hexane whereas the control septa were loaded with 30 µl hexane only. This dose was chosen because an equivalent dose of the aggregation pheromone of *F. occidentalis* had been shown to be biologically active in the field [2], [20].

Pairs of test and control traps with the order randomised within each pair were set out along the length of the rows of eggplants. A series of four trials with six or seven pairs of traps per trial (25 pairs in total) was conducted over 8 days with the spacing between traps within each pair set at either 1.6 m or 4 m and the duration of the trial lasting either 1 day or 4 days. A similar range of trap spacings has been used successfully when testing the aggregation pheromone of *F. occidentalis* (unpublished data). New traps were set out and re-randomised for each of the four trials. The thrips on the traps were identified and sexed under a stereo microscope; the two species could be separated easily by colour, position of wing vein cilia and antennal segment number [16]. The results of the four trials were combined after confirming the absence of a treatment×trial interaction. The data were log_10_(*x*+1) transformed to homogenise the variance and analysed by analysis of variance with trap pairs and trials considered as blocks, using Minitab version 16 (Minitab Inc., USA).

## Results

### Olfactometer Bioassays

Virgin females were attracted to the odour of 50 adult males, but not to the odour of 50 adult females (Table 1). Mixed-age adult females were also attracted to adult males and their preference for the odour side (67%) was similar to that of virgin females (65%). Mixed-age males were also attracted to adult male odours. The preference of mixed-age adult males for adult males (68%) was similar to that of females for males (67%). The proportion of thrips that made no choice within 3 min was low (5% overall, with a range of 3–8% across the four experiments).

**Table 1:** Responses of adult *Thrips palmi* to volatiles produced by 50 adult males or females of the same species in a Y-tube olfactometer.

		Number of choices			
Test insects^a^	Odour source^b^	Odourside	Control side	Preference forodour side (%)^c^	*P* d
Virgin females	Females	29	43	40	0.12
Virgin females	Males	46	25	65	0.017
Females	Males	47	23	67	0.006
Males	Males	49	23	68	0.003

a All test and source insects were adults. Virgin females were 1–3 d post-emergence. Other adults were of mixed age.

b All odour sources consisted of 50 live adults of mixed age.

c The percentage of individual thrips that chose the odour side out of the total that made a choice.

d Exact probability based on null hypothesis of equal preference for the two sides.

### GC/MS of SPME fibres and entrained extracts

**Figure 3:**
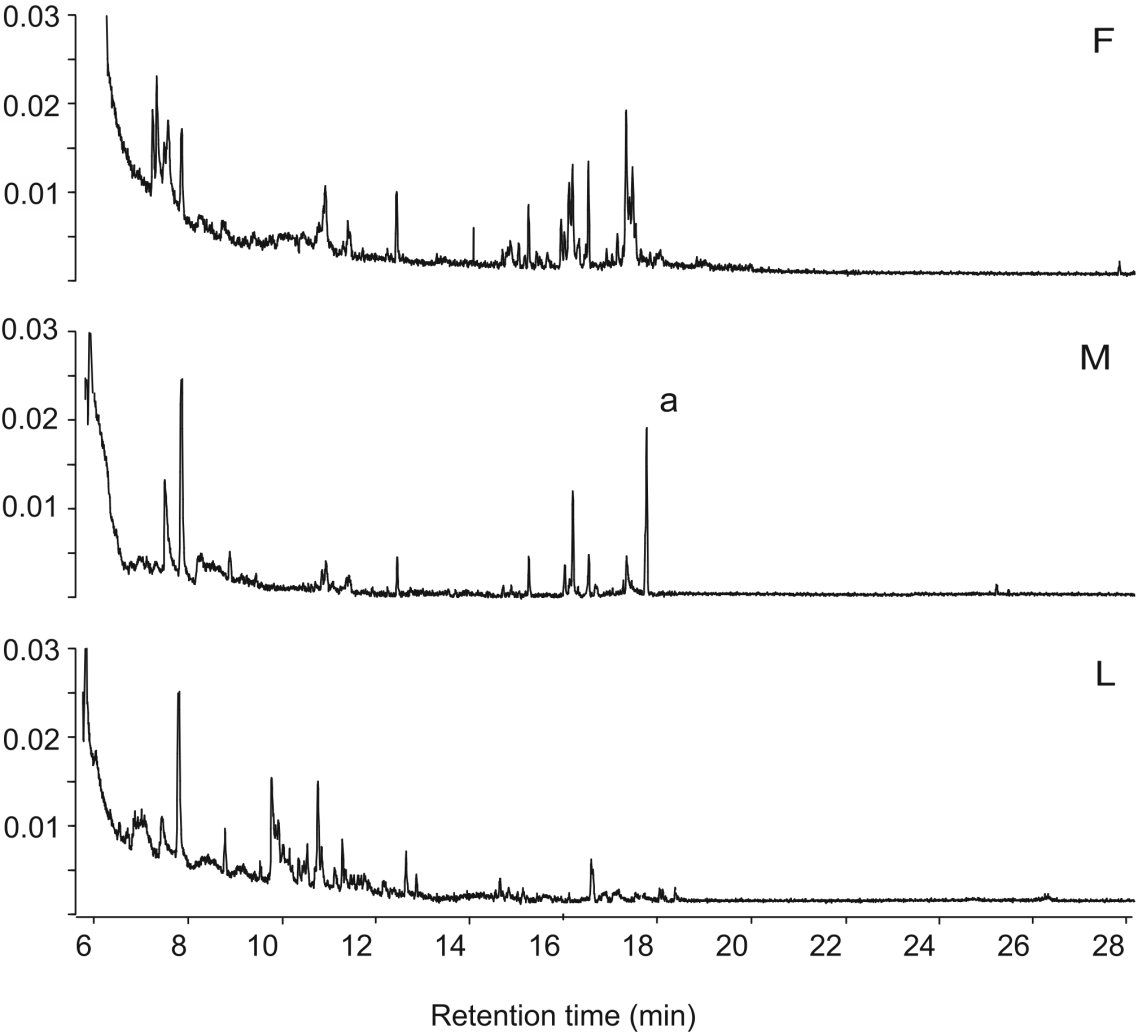
GC/MS traces of SPME-collected headspace volatiles from *Thrips palmi*. GC/MS total ion current (TIC) traces of SPME fibre collections of the headspace volatiles from mixed-age adult females (n = 40) (upper trace F), mixed-age adult males (n = 100) (middle trace M) and larvae (n = 40) (lower trace L) of *Thrips palmi* on a HP5MS column. The major male-specific compound at Rt = 17.65 min in the middle trace is indicated as peak a.

Detailed comparison of TIC chromatograms obtained by GC/MS analysis consistently showed that there was one compound present in SPME fibre entrainments of *T. palmi* adult males that was not present in the females or larvae. Examples of chromatograms of extracts from males, females and larvae are shown in Fig. 3 with the male-specific compound (peak a) present at 17.65 min. The mass spectrum of this compound resembled the mass spectrum of the aggregation pheromone of *F. occidentalis*. Other peaks which were present in males and not in females in the example were not consistently present and are thus likely to be contaminants. Comparison of the area of peak a with a pentadecane standard suggested that it represents approximately 400 pg of material.

In total an estimated 2.2 µg of the male-specific *T. palmi* compound was collected by entrainments of headspace volatiles on Tenax-TA in Japan. A control entrainment in Japan of eggplant material without thrips confirmed that the compound was not produced by eggplants. The EI mass spectrum of the *T. palmi* compound is given in Fig. 2. It was similar to the mass spectrum of the *F. occidentalis* aggregation pheromone, neryl (*S*)-2-methylbutanoate (MW 238) [2] with characteristic ions at m/z 154 (0.4%), 136 (8%), 121 (18%), 93 (61%) and 69 (100%) suggesting a monoterpenoid substructure. An ion at m/z 236 (0.1%) suggested a molecular weight of 236 and ions at m/z 83 (16%) and 55 (75%) suggested the loss of C_4_H_7_CO^+^ and C_4_H_7_^+^ fragments respectively derived from a monounsaturated 5-carbon acid moiety. Isobutane CI analysis gave a strong ion at m/z 237 ([M + H]^+^) confirming the molecular weight as 236. These data suggested that the compound was a monoterpene pentenoate.

The EI mass spectrum and retention index (RI) of the *T. palmi* compound were compared against those of a library of esters of monoterpene alcohols and pentenoic acids. Examples of RIs of three other monoterpene pentenoates are compared with the *T. palmi* compound and lavandulyl 3-methyl-3-butenoate in Table 2. The natural *T. palmi* compound had a retention time identical with that of lavandulyl 3-methyl-3-butenoate on both non-polar (HP5MS) and polar (DBWax) GC columns, and the mass spectra were superimposable. Co-injection of the *T. palmi* compound with lavandulyl 3-methyl-3-butenoate gave peak enhancement on both columns.

**Table 2:** GC retention indices (RI) of natural compound and examples for comparison of synthetic monoterpene pentenoates.

	RI	
	HP5MS	DBWax
natural *Thrips palmi* compound	1525	1854
lavandulyl 3-methyl-3-butenoate	1525	1854
neryl 3-methyl-3-butenoate	1599	1974
geranyl 3-methyl-3-butenoate	1624	2012
chrysanthemyl 3-methyl-3-butenoate(*cis* and *trans*)	1520 & 1530	1838 & 1844

### Chiral Chromatography

The *R* and *S* enantiomers of lavandulyl 3-methyl-3-butenoate gave two partially separated peaks with retention times (Rt) of 45.20 and 45.35 min respectively (Fig. 1A). (*R*)-lavandulyl 3-methyl-3-butenoate gave a single peak with a Rt of 45.19 min (Fig. 1B) with ions at m/z 154 (0.3%), 136 (8%), 121 (18%), 93 (65%), 83 (20%), 81 (10%), 69 (100%) and 55 (76%) and (*S*)-lavandulyl 3-methyl-3-butenoate also gave a single peak at 45.36 min (Fig. 1C). *T. palmi* pheromone eluted at 45.18 min (Fig. 1D), which suggested that the *T. palmi* compound was the *R* enantiomer. Co-injection of the *T. palmi* compound with (*R*)-lavandulyl 3-methyl-3-butenoate standard gave a single peak (Fig. 1E) confirming the *R* configuration for the *T. palmi* compound.

### Field Trials

Test traps caught more *T. palmi* than control traps in all four trials and the effect was statistically significant across the four trials (F_1,24_ = 13.05, *P*<0.001). The results were also significant for females (F_1,24_ = 12.22, *P* = 0.002) and males (F_1,24_ = 6.71, *P* = 0.016) when analysed separately (Table 3).

**Table 3:** Catches of *Thrips palmi* and *Frankliniella intonsa* on blue sticky traps with and without the test compound.

Thrips species	No. on controltraps^a^	No. on test traps^a^	Increase (%)	*P* (test vscontrol)
Trial 1. *Thrips palmi* females	24 (0.62±0.05)	44 (0.82±0.08)	83	-
Trial 2. *Thrips palmi* females	157 (1.43±0.03)	251 (1.60±0.08)	60	-
Trial 3. *Thrips palmi* females	57 (0.97±0.09)	68 (1.06±0.08)	19	-
Trial 4. *Thrips palmi* females	150 (1.37±0.09)	267 (1.64±0.06)	78	-
*Thrips palmi* femalestotal	388 (1.08±0.07)	630 (1.26±0.08)	62	**
Trial 1. *Thrips palmi*males	9 (0.28±0.11)	33 (0.62±0.15)	367	-
Trial 2. *Thrips palmi*males	253 (1.60±0.08)	342 (1.71±0.10)	35	-
Trial 3. *Thrips palmi*males	80 (1.12±0.07)	112 (1.25±0.08)	40	-
Trial 4. *Thrips palmi*males	126 (1.29±0.10)	135 (1.29±0.13)	7	-
*Thrips palmi* malestotal	468 (1.04±0.11)	622 (1.20±0.10)	33	*
*Thrips palmi* total	856 (1.36±0.09)	1252 (1.54±0.09)	46	***
*Frankliniella intonsa* females	96 (0.58±0.07)	117 (0.69±0.05)	22	ns
*Frankliniella intonsa*males	15 (0.12±0.05)	27 (0.18±0.06)	80	ns
*Frankliniella intonsa*total	111 (0.60±0.07)	144 (0.75±0.06)	30	ns

a Total number of individuals caught followed, in brackets, by the mean catch per trap ±SE for the log-transformed data. Note that these standard errors include the variance between trap pairs and also between trials for the analysis of totals. They are therefore not appropriate for comparisons between test and control means. These extra variances are allowed for in the analysis of variance. Response of *Thrips palmi* and *Frankliniella intonsa* to blue sticky traps treated with lures loaded with 30 µg (*R*)-lavandulyl 3-methyl-3-butenoate in 30 µl hexane (test) or 30 µl hexane (control). Key to results of analysis of variance across trials:

*** = *P*<0.001;

** = *P*<0.01;

* = *P*<0.05;

ns = not significant (*P*>0.05); - = statistical comparison not carried out because of small number of replicates within the trial.

There was a trend towards higher numbers of *F. intonsa* on test traps than on control traps in all four trials, but this did not reach statistical significance (F_1,24_ = 3.95, *P* = 0.059), and the results were not significant for females (F_1,24_ = 3.14, *P* = 0.089) and males (F_1,24_ = 1.97, *P* = 0.173) when analysed separately (Table 3). Far fewer thrips of this species were present in the crop and on the traps.

## Discussion

This paper presents the first identification of an aggregation pheromone in the genus *Thrips*. A previous study has shown that an aggregation pheromone was present in another thysanopteran genus *Frankliniella* [2]. Thus aggregation pheromones, which have now been confirmed in two different genera, may be more widespread in this commercially important group of insects than was previously recognised.

The olfactometer bioassays conducted in this study gave behavioural results that were of the same magnitude as those previously obtained with *F. occidentalis*, using similar apparatus [1]. Thus the percentage of *T. palmi* adult males and females that responded positively to the olfactometer arm with the male odour (65–68%) was similar to the percentage recorded in experiments with *F. occidentalis* (66–70%).

In this study we identified the aggregation pheromone of *T. palmi* as the monoterpene pentenoate ester (*R*)-lavandulyl 3-methyl-3-butenoate. The compound was found only in the headspace volatiles collected from males. Its structure was confirmed through comparison of its mass spectrum and three retention indices (RI) (collected on three different GC analytical columns, two achiral and one chiral) with a library of potential monoterpene pentenoate ester matches. Only one compound gave a positive match and this was confirmed by demonstration of peak enhancement. The absolute configuration was confirmed by RI matching with authentic *R* and *S* enantiomers on a chiral analytical GC column and by peak enhancement. The compound was previously obtained serendipitously during the synthesis of the sex pheromone of the vine mealybug *Planococcus ficus* [21].

Significant attraction, in the field, of both female and male *T. palmi* to sticky traps baited with (*R*)-lavandulyl 3-methyl-3-butenoate confirmed the identification of the aggregation pheromone. The pheromone increased trap catches on blue traps, which are already highly visually attractive. The percentage increases (62% females, 33% males) were similar to those found for *F. occidentalis* in experiments in which aggregation pheromone was added at the same dose rate to blue traps in pepper crops under plastic (54% females, 38% males) [2]. These percentage increases in response to pheromone are low compared with those typically obtained in some other insects, such as moths, which can fly upwind for long distances, leading to speculation that the low percentages in thrips could be explained by a missing pheromone component. However, little is known about the role of thrips aggregation pheromones and it cannot be assumed that they act as long-range attractants in the same way as for moths. Higher percentage increases in thrips trap catches can be obtained when pheromones are used in conjunction with less visually attractive traps [22].

The *T. palmi* aggregation pheromone is structurally similar to the aggregation pheromone of *F. occidentalis*. In both cases the compounds are monoterpene esters; in *T. palmi* it is a pentenoate ester i.e. with a double bond in the fatty acid moiety and therefore with a reduced MW of 236, and in *F. occidentalis* it is a pentanoate ester (no extra double bond in the fatty acid moiety) with a MW of 238. In both cases the monoterpene is non-cyclic. Both molecules have chiral centres; in the *T. palmi* molecule the chiral centre is present in the monoterpene moiety and in the *F. occidentalis* molecule the chiral centre is present in the fatty acid moiety. The overall similarities are surprising because although *T. palmi* and *F. occidentalis* belong to the same sub-family Thripinae of the family Thripidae, they belong to two major and distinct groups: the *Thrips* genus-group and the *Frankliniella* genus-group. These are considered to be only distantly related [23], probably having separated and diversified about 80–120 million years ago [24].

The source of the aggregation pheromone is unknown for *T. palmi* and other species. The sternal glands [6] are a possible source [5], but this remains to be confirmed. In experiments with *F. occidentalis*, the aggregation pheromone and the recently discovered contact pheromone, 7-methyltricosane (C_24_H_50_), were extracted from the surface over which male *F. occidentalis* moved [25]. The sternal glands, on the underside of the abdomen, would be conveniently positioned for depositing these chemicals.

The proportion of male *T. palmi* caught on the pheromone traps or control traps (50–55%) was markedly higher than the proportion of males found on the crop when collecting manually (5–8%). This phenomenon has been recorded before in *T. palmi* [26], *F. intonsa* [27] and *F. occidentalis* [28] and has been attributed to the greater activity of males [28].

The global trade in plants and plant products increases the risk of expansion of the range of *T. palmi* and the frequency of outbreaks within its current range. Where expansion does occur, control and eradication has been achieved so far, but it is recognised that successful management can be greatly facilitated by early intervention [9], [15]. European Plant Health authorities are seeking improved methods for early detection as part of their contingency planning and deployment of pheromone-baited traps has been proposed as a potentially effective technique. Mass trapping experiments have confirmed this potential; field deployment of the aggregation pheromone of *F. occidentalis* doubled the catch on blue attractive traps [29], illustrating the possibility of using the technique to achieve earlier detection of the small populations typically present soon after the introduction of quarantine thrips.

In addition, field use of *F. occidentalis* aggregation pheromone in conjunction with the attractive traps, resulted in a combined reduction of 73% in thrips numbers and 68% in damage to strawberry crops, showing that in contained environments the approach can make a cost-effective contribution to population reduction in high-value crops, although further development work is required to achieve a more consistent outcome in commercial production systems [29]. The use of a thrips aggregation pheromone as part of an IPM programme has the advantages of removing both females and males, and may reduce both the rate of development of insecticide resistance and insecticide residues on crops.

Thus the enhanced trap catch of *T. palmi* provided by this aggregation pheromone may be a useful component of future pest management approaches, supporting improved quarantine detection, monitoring and control.
